# Cu_2_S Nanoflakes Decorated with NiS Nanoneedles for Enhanced Oxygen Evolution Activity

**DOI:** 10.3390/mi13020278

**Published:** 2022-02-09

**Authors:** Le Wang, Mancong Li, Yingxin Lyu, Jiawen Liu, Jimin Du, Dae Joon Kang

**Affiliations:** 1 College of Chemistry and Molecular Engineering, Zhengzhou University, Zhengzhou 450001, China; wl3200852214@163.com (L.W.); lmc1234562022@163.com (M.L.); 2 School of Chemistry and Chemical Engineering, Anyang Normal University, Anyang 455002, China; l2801106022@163.com (Y.L.); l718372708@163.com (J.L.); 3 Department of Physics, Sungkyunkwan University, 2066, Seobu-ro, Jangan-gu, Suwon 16419, Gyeonggi-do, Korea

**Keywords:** NiS, Cu_2_S, electrocatalysts, oxygen evolution reaction

## Abstract

Metal sulfides are considered excellent materials for oxygen evolution reaction because of their excellent conductivity and high electrocatalytic activity. In this report, the NiS-Cu_2_S composites were prepared on copper foam (NiS-Cu_2_S-CF) using a facile synthetic strategy. The scanning electron microscopy results confirmed that the NiS nanoneedles were successfully grown on Cu_2_S nanoflakes, greatly increasing the active sites. Particularly, the optimized 15% NiS-Cu_2_S-CF composite demonstrated excellent oxygen evolution activity with a small overpotential of 308 mV@20 mA cm^−2^, which is significantly smaller than that of noble metal-based electrocatalysts and other NiS-Cu_2_S-CF composites. The enhanced oxygen evolution activity is attributed to the unique morphology that can provide ample active sites, rich ion-transfer pathways, and the synergistic effect between NiS and Cu_2_S, which can boost the electron transfer rate.

## 1. Introduction

Nowadays, precious metal-based materials, including RuO_2_ and IrO_2_, are considered excellent electrocatalysts for oxygen evolution reactions (OER) [1,2]. However, their scarcity and high cost prevent them from being used on a large scale. Hence, the development of earth-abundant, low-cost, high-performance catalysts to replace precious-metal-based electrocatalysts has become critical. Because of their high electrical conductivity, excellent catalytic performance, ease of synthesis, and low cost, metal sulfide-based materials have received much attention [3,4,5,6,7,8]. Of the various metal sulfides, Cu_2_S is considered a potential material for oxygen evolution because of its distinct morphology, good conductivity, and high catalytic performance [9,10,11]. Many researchers have prepared Cu_2_S and its composites for OER [12,13,14,15]. However, the OER activities of the Cu_2_S-based materials are still poor compared to those of precious metal-based materials.

Meanwhile, NiS has recently been demonstrated to be an excellent cocatalyst for oxygen evolution because of its good chemical and physical properties [16,17,18]. Furthermore, the construction of binary metal sulfides heterostructure is a promising route for significantly improving the OER activity of catalysts because the heterostructure can enhance charge transfer and provides more surface-active sites [19,20,21]. For instance, Li et al. fabricated the NiS/Bi_2_WO_6_ heterostructure for OER, which exhibited a small overpotential of 527 mV@10 mA cm^−2^ [22]. Luo et al. synthesized the NiS/C_3_N_4_ composites for oxygen evolution, which displayed a small overpotential of 334 mV@10 mA cm^−2^ [23]. Jiang et al. constructed a NiS/Fe_3_O_4_ composite with a small overpotential of 310 mV@10 mA cm^−2^ for OER [24]. As a result, taking into account the exceptional catalytic performance of the NiS, we attempted to integrate the NiS with Cu_2_S to construct NiS-Cu_2_S heterostructure, which could be an efficient strategy for increasing the OER activity of the Cu_2_S. Hence, we rationally designed NiS nanoneedles on Cu_2_S nanoflakes as an efficient OER electrocatalyst, which has not yet been reported.

In addition, electrocatalysts grown on 3D porous copper foam (CF) can provide not only a large surface area but also abundant ion-transfer pathways in the OER process. Hence, we employed CF as a 3D porous template substrate in this work. The NiS-Cu_2_S composites were prepared on CF (NiS-Cu_2_S-CF) using a facile synthetic route. The scanning electron microscopy (SEM) images demonstrated that the NiS nanoneedles were successfully grown on Cu_2_S nanoflakes, providing a large number of active surface sites. The electrochemical measurement results indicated that the optimized 15% NiS-Cu_2_S-CF electrocatalysts exhibited an outstanding OER activity of 308 mV at 20 mA cm^−2^ in a 1 M KOH electrolyte, which is much higher than that of RuO_2_-based electrocatalysts (350 mV at 20 mA cm^−2^). These results suggest that the NiS-Cu_2_S-CF composite be a candidate for oxygen evolution.

## 2. Materials and Methods

Fabrication of NiS-Cu_2_S heterostructure on CF: the NiS-Cu_2_S heterostructure was prepared through a facial synthetic route combined with electrochemical-corrosion and hydrothermal reaction methods. More detailed information about the preparation process, materials characterizations, and electrocatalytic performance test are provided in Supporting Information.

## 3. Results

**Figure 1:**
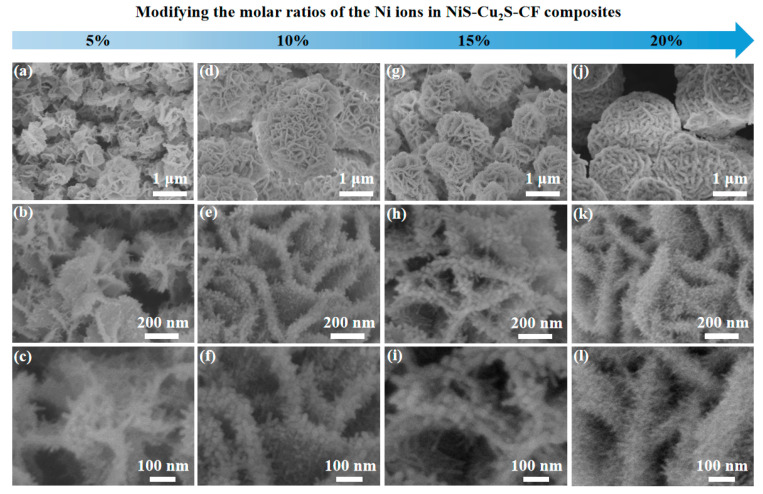
The SEM images of the as-prepared samples. (**a**–**c**) 5% NiS-Cu_2_S-CF composites, (**d**–**f**) 10% NiS-Cu_2_S-CF composites, (**g**–**i**) 15% NiS-Cu_2_S-CF composites, and (**j**–**l**) 20% NiS-Cu_2_S-CF composites.

The fabrication process of the NiS-Cu_2_S-CF composites is given in the following section. Firstly, the Cu_2_O was fabricated on CF using the electrochemical-corrosion method; then, the Cu_2_S-CF was formed by the sulfidation of Cu_2_O-CF; finally, the NiS was grown on Cu_2_S-CF by a facile hydrothermal method. More detailed information on the fabrication process is shown in Supporting Materials. To improve the OER performance of the sample, we tuned the molar ratios of Ni and Cu in the NiS-Cu_2_S-CF composites. The effects of molar ratios of Ni and Cu in the NiS-Cu_2_S-CF composites were investigated through an inductively coupled plasma atomic emission spectrometer (ICP-AES). The result is displayed in Table S1 (Supporting Materials). The SEM was employed to examine the morphology of the as-prepared products. Figure S1 (Supporting Materials) show the SEM images of the CF and Cu_2_O-CF. Figure S2 (Supporting Materials) shows the SEM image of Cu_2_S-CF composites, indicating that the nanoflake-shaped Cu_2_S were successfully grown on CF. Such morphology can offer a large number of growth sites for NiS. Figure S3 (Supporting Materials) presents the SEM image of NiS-CF composites showing the NiS with nanoneedles were successfully decorated on CF. The SEM images of the NiS-Cu_2_S-CF composites are shown in Figure 1. As shown, when the molar ratio of the Ni ions was 5%, the surface of the Cu_2_S nanoflakes was found to be decorated with a limited amount of NiS nanoneedles (Figure 1a–c). When the molar ratio of the Ni ions approached 10% and 15%, numerous NiS nanoneedles were uniformly grown on the Cu_2_S surface (Figure 1d–i). The length and diameter of the NiS nanoneedles were 40 and 10 nm, respectively. Furthermore, as the molar ratio of the Ni ions reaches 20%, the size of NiS decreased, possibly because a large amount of the Ni ions affected the length of the NiS nanoneedles (Figure 1j–l). Here, we also propose a plausible formation mechanism on how the NiS nanoneedles were formed on Cu_2_S nanoflakes; once Ni ions were evenly distributed on the surface of Cu_2_S nanoflakes, a large amount of NiS nuclei were formed on the Cu_2_S nanoflakes during the hydrothermal process. NiS nanoneedles were successfully grown on Cu_2_S nanoflakes with an increase in reaction time. It should be noted that the Cu_2_S nanoflakes decorated with NiS nanoneedles can not only provide numerous surface active sites, but can also offer more contact areas between electrodes and electrolytes during the oxygen evolution process.

**Figure 2:**
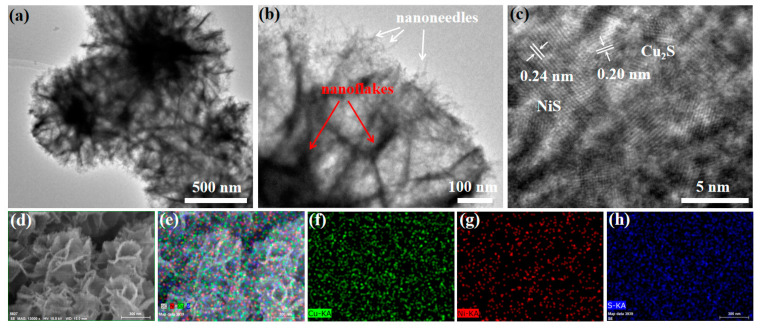
(**a**,**b**) TEM and (**c**) HRTEM images of the 15% NiS-Cu_2_S-CF composites. (**d**–**h**) EDS mappings of the 15% NiS-Cu_2_S-CF composites verifying the presence of Cu, Ni, and S elements.

The transmission electron microscopy (TEM) result presents the NiS-Cu_2_S composites with a flake structure (Figure 2a). The enlarged TEM image (Figure 2b) exhibits the surface of the Cu_2_S decorated with many NiS nanoneedles. The high-resolution TEM (HRTEM) image displays the two distinctive types of lattice fringes with the neighboring distance of 0.20 and 0.24 nm (Figure 2c), which correspond to the (601) facet of Cu_2_S and (220) plane of NiS, respectively. Furthermore, these two lattice fringes are well-connected, indicating that the high-quality heterostructure was formed between NiS and Cu_2_S, resulting in a significant improvement in the structural stability of the catalysts [25,26]. This is due to the following reasons: (1) the formed heterostructure can significantly increase the ion transfer rate between NiS and Cu_2_S; (2) the NiS nanoneedles grown on Cu_2_S reduce the ion-transfer distance between electrolyte and electrode, and reduce the corrosion time of the sample during the OER process, and improve the structural stability [27]. The energy-dispersive X-ray spectroscopy (EDS) elemental mappings of the 15% NiS-Cu_2_S-CF and other samples (5, 10, and 20% NiS-Cu_2_S-CF) are shown in Figure 2d–h and Figures S4–S6 (Supporting Materials), indicating that Cu, Ni, and S elements are uniformly distributed in the fabricated electrocatalysts.

**Figure 3:**
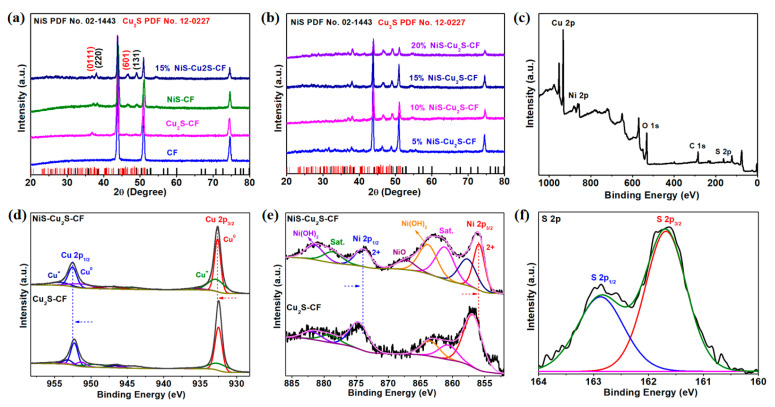
(**a**,**b**) XRD patterns of the as-prepared electrocatalysts. € (**c**) The fully XPS spectra of 15% NiS-Cu_2_S-CF composites. (**d**) high-resolution XPS spectra of Cu 2p in NiS-Cu_2_S-CF and Cu_2_S-C€ (**e**) high-resolution XPS spectra of Ni 2p in NiS-Cu_2_S-CF and NiS-CF. (**f**) high-resolution XPS spectra of S 2p in NiS-Cu_2_S-CF.

Figure 3a,b show the X-ray diffractometry (XRD) patterns of the as-prepared products. As displayed in Figure 3a, for Cu_2_S-CF composites, two peaks at 36.75° and 46.50° belong to the (0111) and (601) planes of the Cu_2_S (JCPDS No. 12-0227) [28]. For NiS-CF composites, two peaks at 37.79° and 48.95° can be assigned to the (220) and (131) planes of NiS (JCPDS No. 02-1443) [29]. Furthermore, the CF substrate presents three diffraction peaks at 43.90°, 50.95°, and 74.61°. It should be noted that, with the increase in the amount of the Ni ions, the peak’s intensity of the (220) and (131) of the NiS becomes increased, which suggests the large amount of NiS was successfully grown on the Cu_2_S. Furthermore, the chemical composition and electronic interaction of the samples were measured by X-ray photoelectron spectroscopy (XPS). Figure 3c displays the full XPS spectra of 15% NiS-Cu_2_S-CF composites, indicating the presence of S, Cu, and Ni elements. The XPS spectra of Cu 2p in NiS-Cu_2_S-CF (Figure 3d) display two peaks at 932.4 and 952.2 eV along with two satellite peaks corresponding to the Cu^0^ 2p_3/2_, Cu^+^ 2p_3/2_, Cu^0^ 2p_1/2_, and Cu^+^ 2p_1/2_, respectively [30]. In addition, the XPS spectra of Ni 2p in NiS-Cu_2_S-CF (Figure 3e) show two peaks at 856.1 (Ni^2+^ 2p_3/2_) and 873.8 eV (Ni^2+^ 2p_1/2_) along with two satellite peaks at 862.8 and 881.3 eV [31]. Moreover, the Ni(OH)_2_ (863.92 and 881.52 eV) and NiO (867.36 eV) peaks were observed, confirming the oxidation state of the NiS. Compared to Cu_2_S-CF and NiS-CF, the Cu 2p and Ni 2p of 15% NiS-Cu_2_S-CF show positive and negative shifts, respectively, confirming the electronic interaction between NiS and Cu_2_S. Furthermore, such shifts in the Cu and Ni spectra revealed strong interfacial electron transfer from Cu_2_S to NiS caused by the high electronegativity of Ni atoms [32]. It should be noted that such unique electronic interactions will promote the charge transfer between NiS and Cu_2_S and increase the OER performance [33]. The S 2p spectra of the NiS-Cu_2_S-CF, Cu_2_S-CF, and NiS-CF are shown in Figure 3f and Figure S7 (Supporting Materials), which confirm the presence of S ions in the fabricated samples [34,35]. In addition, the XPS of 5, 10, and 20% NiS-Cu_2_S-CF composites are presented in Figures S8–S10 in Supporting Materials, which also confirm the oxidation state of the NiS.

**Figure 4:**
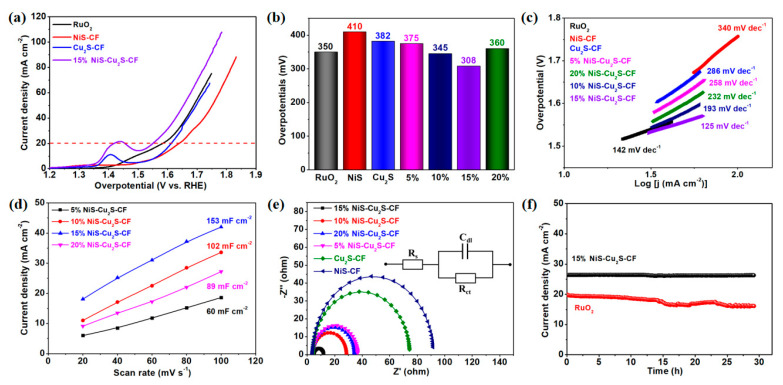
(**a**) LSV curves of the RuO_2_, NiS-CF, Cu_2_S-CF, and 15% NiS-Cu_2_S-CF. (**b**) The overpotentials of the as-fabricated samples at the current density of 20 mA cm^−2^. (**c**) TF slope of the as-fabricated samples. (**d**) *C_dl_* of the as-fabricated samples. (**e**) EIS of the as-fabricated samples (the inset shows the equivalent circuit). (**f**) Long-term cycling stability of 15% NiS-Cu_2_S-CF and RuO_2_.

We employed the as-prepared NiS-Cu_2_S-CF composites as the electrocatalyst for oxygen evolution in 1 M KOH electrolyte. Figure 4a and Figure S11 (Supporting Materials)  show the linear sweep voltammetry (LSV) curves of the prepared samples. As demonstrated, the 15% NiS-Cu_2_S-CF composite shows superior OER activity to those of pure CF, RuO_2,_ and other NiS-Cu_2_S-CF composites. Figure 4b presents the overpotentials of the as-prepared samples at the current density of 20 mA cm^−2^. Note that the 15% NiS-Cu_2_S-CF composite showed a small overpotential of 308 mV at 20 mA cm^−2^. Furthermore, the overpotential of the 15% NiS-Cu_2_S-CF composite (~420 mV) was smaller than that of RuO_2_ (455 mV) when the current density reached up to 50 mA cm^−2^. The superior catalytic activity could be attributed to the following reasons: (1) the unique morphology can provide more ion-transfer pathways; (2) the NiS nanoneedles can provide ample reaction active sites; (3) the Cu_2_S with high conductivity can promote the charge transfer ability; and (4) the synergistic effect between NiS and Cu_2_S will increase the catalytic property. In addition, the overpotential of the 15% NiS-Cu_2_S-CF composite was much lower than those previously reported (Table 1) [10,12,13,15,22,23,24,36,37,38,39], suggesting that the as-fabricated NiS-Cu_2_S-CF composite is a promising OER electrocatalyst. Figure 4c shows the Tafel slopes of the as-prepared electrocatalysts. The Tafel slopes of RuO_2_, NiS-CF, Cu_2_S-CF, 5% NiS-Cu_2_S-CF, 10% NiS-Cu_2_S-CF, 15% NiS-Cu_2_S-CF, and 20% NiS-Cu_2_S-CF are approximately 142, 340, 286, 258, 193, 125, and 232 mV dec^−1^, respectively. The 15% NiS-Cu_2_S-CF composite shows a lower value of the Tafel slope, indicating that it has rapid catalytic kinetics during the OER process [40]. It is widely accepted that the electrochemically active surface area has an important effect on the OER. Hence, the cyclic voltammetry (CV) curve was taken to assess the electrochemical double-layer capacitance (*C_dl_*) of the samples. The CV curves at various scan rates of the as-prepared samples are shown in Figure S12 (Supporting Materials). The calculated *C_dl_* of the samples is shown in Figure 4d, and the *C_dl_* values of 5% NiS-Cu_2_S-CF, 10% NiS-Cu_2_S-CF, 15% NiS-Cu_2_S-CF, and 20% NiS-Cu_2_S-CF are approximately 60, 102, 153, and 89 mF cm^−2^, respectively. The 15% NiS-Cu_2_S-CF has the highest value of the *C_dl_*, demonstrating that it can provide more catalytic active sites during the OER process [41,42]. The electrochemical impedance spectroscopy (EIS) was performed at the potential of 1.51 V vs. reversible hydrogen electrode (RHE) to investigate the charge transfer performance of the samples. Figure 4e presents the EIS spectra and the equivalent circuit (the inset of Figure 4e) of the samples, indicating that the charge-transfer resistance (*R_ct_*) of NiS-CF, Cu_2_S-CF, 5% NiS-Cu_2_S-CF, 10% NiS-Cu_2_S-CF, 15% NiS-Cu_2_S-CF, and 20% NiS-Cu_2_S-CF is 87.1, 70.3, 32.9, 24.5, 8.2, and 30.7 Ω, respectively. The 15% NiS-Cu_2_S-CF composite shows a smaller *R_ct_*, indicating a faster ion-transfer rate during the OER process than other samples [43,44,45]. The smaller R*_ct_* of 15% NiS-Cu_2_S-CF is due to the appropriate amount of NiS grown on CuS, which can greatly enhance the electrical conductivity, resulting in a higher charge transfer rate between the electrode and electrolyte. Moreover, we also tested the cycling stability of the 15% NiS-Cu_2_S-CF composite. Figure S13 (Supporting Materials) presents the LSV curves of the 15% NiS-Cu_2_S-CF composite before and after 1000 cycling tests. The LSV curves slightly changed after the 1000 cycling tests, which indicates that the 15% NiS-Cu_2_S-CF composite has excellent cycling catalytic activity. Figures S14 and S15 (Supporting Materials) show the SEM and XPS results of the 15% NiS-Cu_2_S-CF composite after 1000 cycling tests. The SEM image indicates that the 15% NiS-Cu_2_S-CF composite shows no appreciable structure change compared to before the cycling test. The XPS results indicate the presence of a metal-oxygen (M-O) bond after 1000 cycling tests (Figure S15e), indicating that 15% NiS-Cu_2_S-CF was oxidated during the cycling tests. Furthermore, the long-term cycling stability of the 15% NiS-Cu_2_S-CF and RuO_2_ was measured using the amperometric *i–t* curve at a potential of 1.53 V vs. RHE. As shown in Figure 4f, even tested for 30 h, the 15% NiS-Cu_2_S-CF composite still shows a remarkable OER activity than RuO_2_. This result confirms that our NiS-Cu_2_S-CF composite is a stable and cost-effective electrocatalyst for OER.

## 4. Conclusions

In conclusion, the NiS-Cu_2_S-CF composites with a unique morphology were successfully prepared using a facile method. The optimized 15% NiS-Cu_2_S-CF electrocatalyst exhibited a lower overpotential of 308 mV at 20 mA cm^−2^ for OER than that of noble-metal-based electrocatalysts, and those metal sulfides reported previously. The composite’s remarkable catalytic performance is attributed to (1) its unique morphology that provides not only more reaction active sites but also more ion-transfer pathways; (2) improved conductivity and enhanced structural stability of the formed NiS-Cu_2_S heterostructure; (3) increased electron transfer property owing to the synergistic effect between NiS and Cu_2_S. This work demonstrates that the NiS-Cu_2_S-CF composite is a potential candidate for high-performance oxygen evolution.

## Figures and Tables

**Table 1:** Comparison of the electrocatalytic activity of the metal sulfide-based electrocatalysts for OER.

Electrocatalysts	Overpotential (mV)	Current Density (mA cm^−2^)	Ref.
15% NiS-Cu_2_S-CF	308	20	This work
Cu_2_S-CF	336	20	[10]
CuS-CC	358	10	[12]
Cu_2_S-Ni(OH)_2_	500	10	[13]
Cu_2_S-CuO	380	10	[15]
NiS-Bi_2_WO_6_	527	10	[22]
NiS-C_3_N_4_	334	10	[23]
NiS-Fe_3_O_4_	310	10	[24]
NiS-Fe_3_S_4_	338	10	[36]
NiS_x_-Fe_3_O_4_-rGO	330	10	[37]
Cu_9_S_5_-Ni foam	301	10	[38]
3% Ni-doped CuS	390	10	[39]

## Data Availability

All data needed to evaluate the conclusions in the paper are present in the paper and/or the Supplementary Materials. Additional data related to this paper may be requested from the authors.

## Supplementary Materials

The following supporting information can be downloaded at: https://www.mdpi.com/article/10.3390/mi13020278/s1, fabrication of electrocatalysts on copper foam, materials, and electrochemical characterizations, Table S1: Molar ratios of Ni and Cu ions in the NiS-Cu_2_S-CF composites; Figure S1: SEM images of the (a) CF and (b) Cu_2_O-CF composites; Figure S2: SEM images of the Cu_2_S-CF composites; Figure S3: SEM images of the NiS-CF composites; Figure S4: The S XPS spectra of the (a) Cu_2_S-CF and (b) NiS-CF composites; Figure S5: LSV curves of the 5% NiS-Cu_2_S-CF, 10% NiS-Cu_2_S-CF, and 20% NiS-Cu_2_S-CF composites; Figure S6: CV curves of the (a) 5% NiS-Cu_2_S-CF composites, (b) 10% NiS-Cu_2_S-CF composites, (c) 15% NiS-Cu_2_S-CF composites, and (d) 20% NiS-Cu_2_S-CF composites; Figure S7: LSV curves of the 15% NiS-Cu_2_S-CF composites after 1000 cycles test; Figure S8: SEM images of the 15% NiS-Cu_2_S-CF composites after 1000 cycles test; Figure S9: XPS of the 15% NiS-Cu_2_S-CF composites after 1000 cycles test. (a) Fully XPS spectra, (b) Cu 2p, (c) Ni 2p, (d) S 2p, and (e) O 1s.
