# Supplementary material for: Cu2S Nanoflakes Decorated with NiS Nanoneedles for Enhanced Oxygen Evolution Activity
Source: Micromachines (Basel). 2022 Feb 9;13(2):278. doi: 10.3390/mi13020278 (PMC8875390; doi:10.3390/mi13020278)
Supplement: Supplementary file 1 [file micromachines-13-00278-s001.zip › micromachines-1575580-supplementary.pdf]

Supporting Information

Cu_2S nanoflakes decorated with NiS nanoneedles for enhanced oxygen evolution activity

Le Wang, Mancong Li, Yingxin Lyu, Jiawen Liu, Jimin Du and Dae Joon Kang

Experimental Section

Fabrication of Cu_2S on copper foam

The Cu_2S was synthesized on copper foam (CF) by using a simple method. A typical synthesis procedure is given as follows. A piece of CF (2.5 cm × 2.5 cm) was cleaned with methanol, ethanol, and distilled (DI) water for 30 min each. The CF was then dried in a vacuum at 60 °C for 12 h. The Cu_2O was first deposited on the CF by an electrochemical-corrosion process using the CHI 660E electrochemical workstation in 1 M KOH. The Hg/HgO and a piece of CF were used as the reference and working electrodes. Another piece of CF was used as the counter electrode. The Cu_2O was deposited on CF (Cu_2O-CF) by chronopotentiometry under a current density of 10 mA cm^{-2} for 10 min. The Cu_2O-CF was cleaned several times with DI water and ethanol. In addition, a hydrothermal process was used to form Cu_2S-CF composites. 3 mmol of thiourea was added into 25 mL of DI water under magnetic stirring. The above mixture and Cu_2O-CF composites were then placed into an autoclave and kept at 160 °C for 24 h. Finally, the product was cleaned several times with DI water and ethanol, before being dried in a vacuum at 65 °C for 10 h.

Fabrication of NiS-Cu_2S Heterostructure on Copper Foam

The NiS was grown on Cu_2S-CF through a hydrothermal approach. To prepare 5% NiS-Cu_2S-CF electrocatalysts (where 5% is the molar ratio of Ni to Cu ions), at first. 0.05 mmol nickel acetate and 0.3 mmol thioureas were added into 25 mL DI water and stirred for 20 min. The above mixture and a piece of Cu_2S-CF composites were then placed into an autoclave and kept at 160 °C for 24 h. Finally, the product was cleaned several times with DI water and

ethanol, and dried in a vacuum at 65 °C for 10 h. The same method was used to prepare 10% NiS-Cu_2S-CF, 15% NiS-Cu_2S-CF, 20% NiS-Cu_2S-CF, and NiS-CF electrocatalysts by modifying the molar ratio of nickel acetate and thiourea.

Materials Characterization

A field-emission scanning electron microscope (HITACHI-SU8010) was used to examine the morphology of the as-prepared electrocatalyst. The crystal phases of the fabricated electrocatalyst were characterized using a powder X-ray diffractometer (Rigaku Ultima III) with Cu K_α radiation (λ = 1.5418 Å). The microstructure was examined using a transmission electron microscope (FEI Tecnai G2 F20) operated at 200 kV. XPS was performed using an ESCALAB 250 Xi. The molar ratios of Ni and Cu in the NiS-Cu_2S-CF composites were tested using an inductively coupled plasma atomic emission spectrometer (ICP-AES, Optima 7300 DV, Perkin Elmer Co., USA).

Electrochemical characterizations

A three-electrode system was used for electrochemical characterization in 1 M KOH with the CHI 660E electrochemical workstation. The reference and counter electrodes were prepared of Hg/HgO and a graphite rod respectively. The working electrode was made from the fabricated samples. Linear-sweep voltammetry (LSV) was carried out in KOH solution with a scan rate of 2 mV s^{-1}. All the LSV curves were treated by *iR* correction. The potential used in this work was referenced to a reversible hydrogen electrode (RHE) potential using the following equation: $E_{RHE} = E_{Hg/HgO}$ + 0.098 V + 0.059 PH. Cyclic voltammetry (CV) was carried out at scan rates of 20, 40, 60, 80 and 100 mV s^{-1} to investigate the electrochemically active surface area. EIS was performed at a frequency ranging from 0.1 to 100 kHz, and 1,000-cyclic CV measurement was carried out in the range from 0.1 to 0.8 V *vs.* RHE at 50 mV s^{-1} to characterize the catalytic stability. The long-term stability was tested by measuring amperometric *i–t* curves. To compare the OER performance of RuO_2 powder, a RuO_2 catalyst was constructed by dispersing 5 mg of RuO_2 in 750 μL DI water with 200 μL ethanol and 50

μL Nafion (5 wt%). 10 μL of the ink sample was then dropped onto a piece of CF and dried at room temperature.

Table S1. Molar ratios of Ni and Cu ions in the NiS-Cu_2S-CF composites.

Electrocatalysts	Molar ratio of Ni ions	Molar ratio of Cu ions
5% NiS-Cu_2S-CF	7 μmol cm^{-2} (0.4 mg cm^{-2})	95 μmol cm^{-2} (9.1 mg cm^{-2})
10% NiS-Cu_2S-CF	12 μmol cm^{-2} (0.9 mg cm^{-2})	96 μmol cm^{-2} (9.2 mg cm^{-2})
15% NiS-Cu_2S-CF	20 μmol cm^{-2} (1.3 mg cm^{-2})	95 μmol cm^{-2} (9.1 mg cm^{-2})
20% NiS-Cu_2S-CF	26 μmol cm^{-2} (1.8 mg cm^{-2})	95 μmol cm^{-2} (9.1 mg cm^{-2})

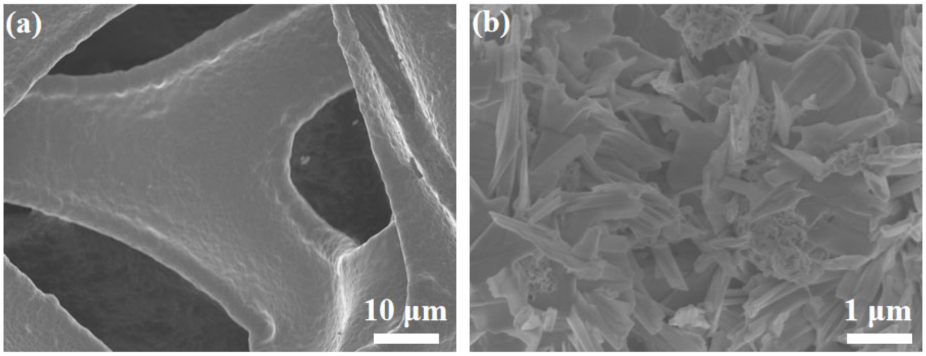

Figure S1. SEM images of the (a) CF and (b) Cu_2O-CF composites.

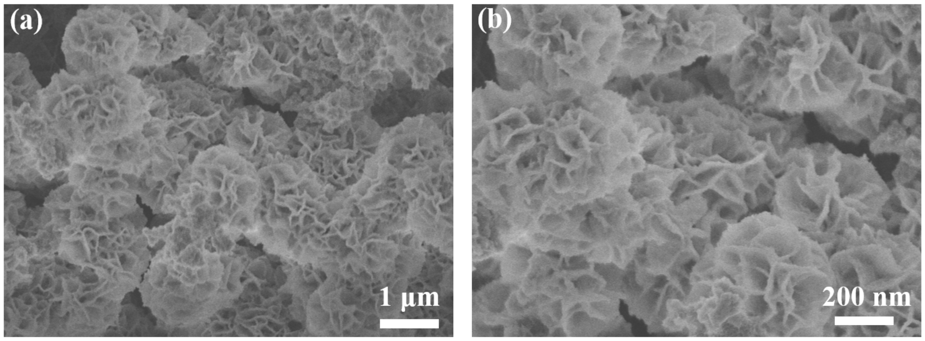

Figure S2. (a,b) SEM images of the Cu_2S-CF composites.

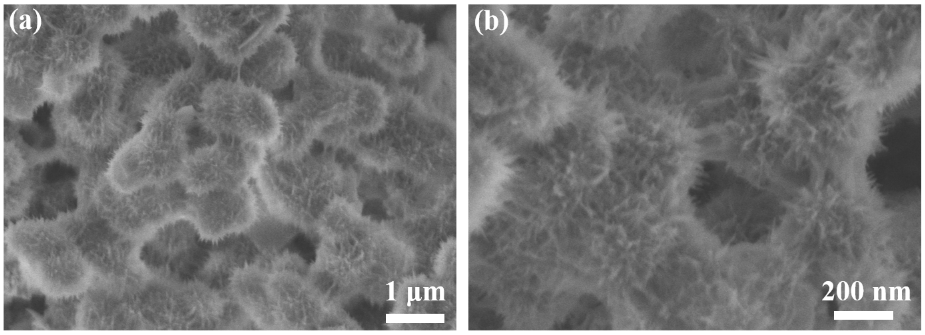

Figure S3. (a,b) SEM images of the NiS-CF composites.

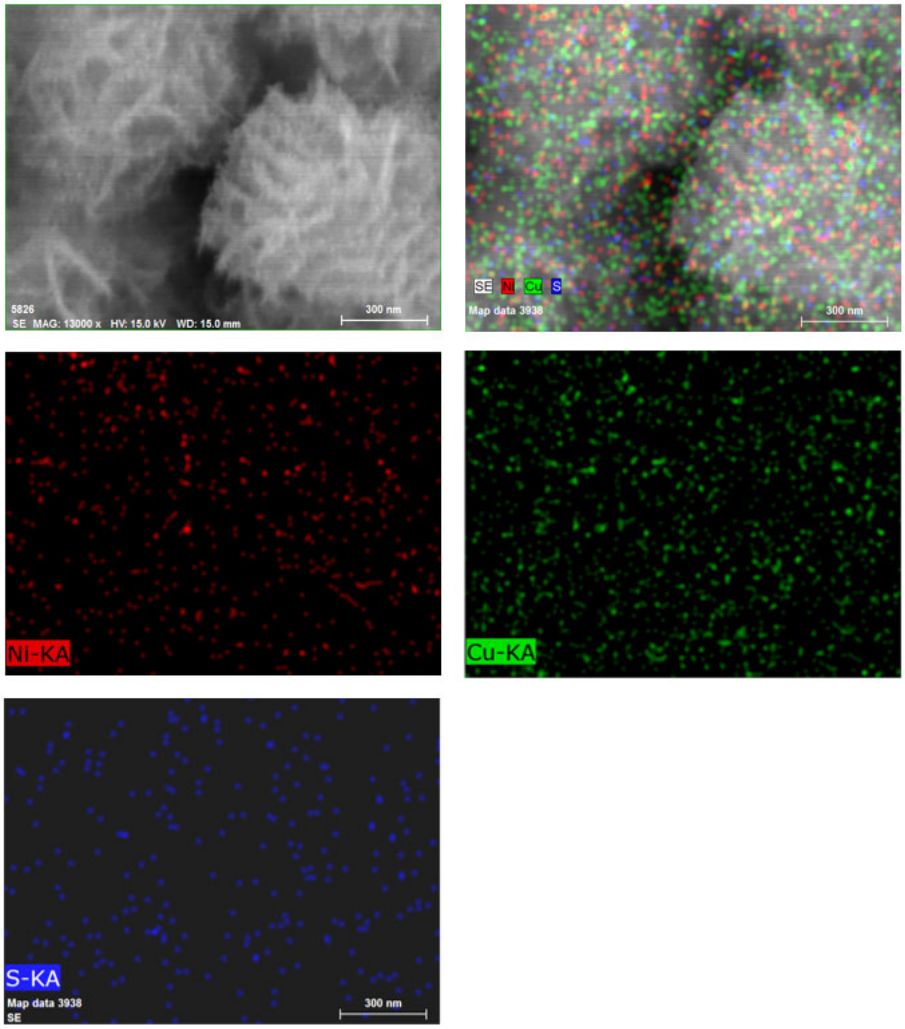

Figure S4. EDS elemental mapping of the 5% NiS-Cu_2S-CF composites.

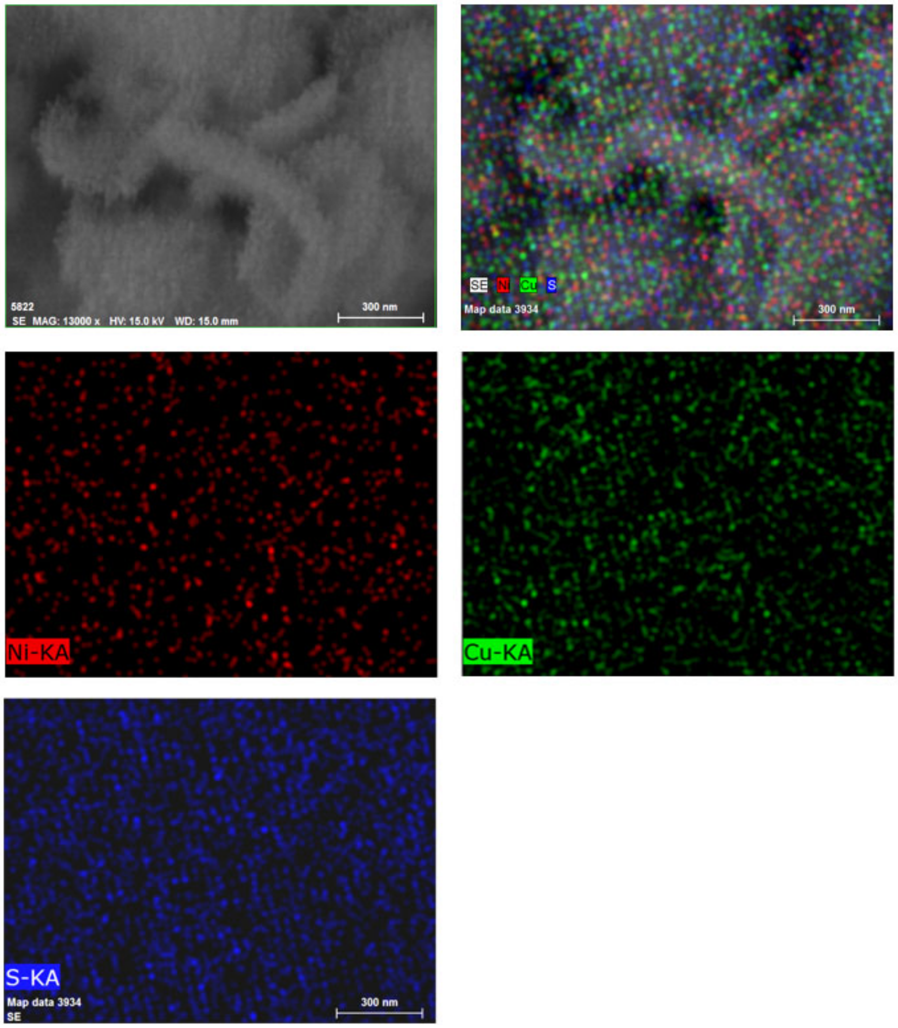

Figure S5. EDS elemental mapping of the 10% NiS-Cu_2S-CF composites.

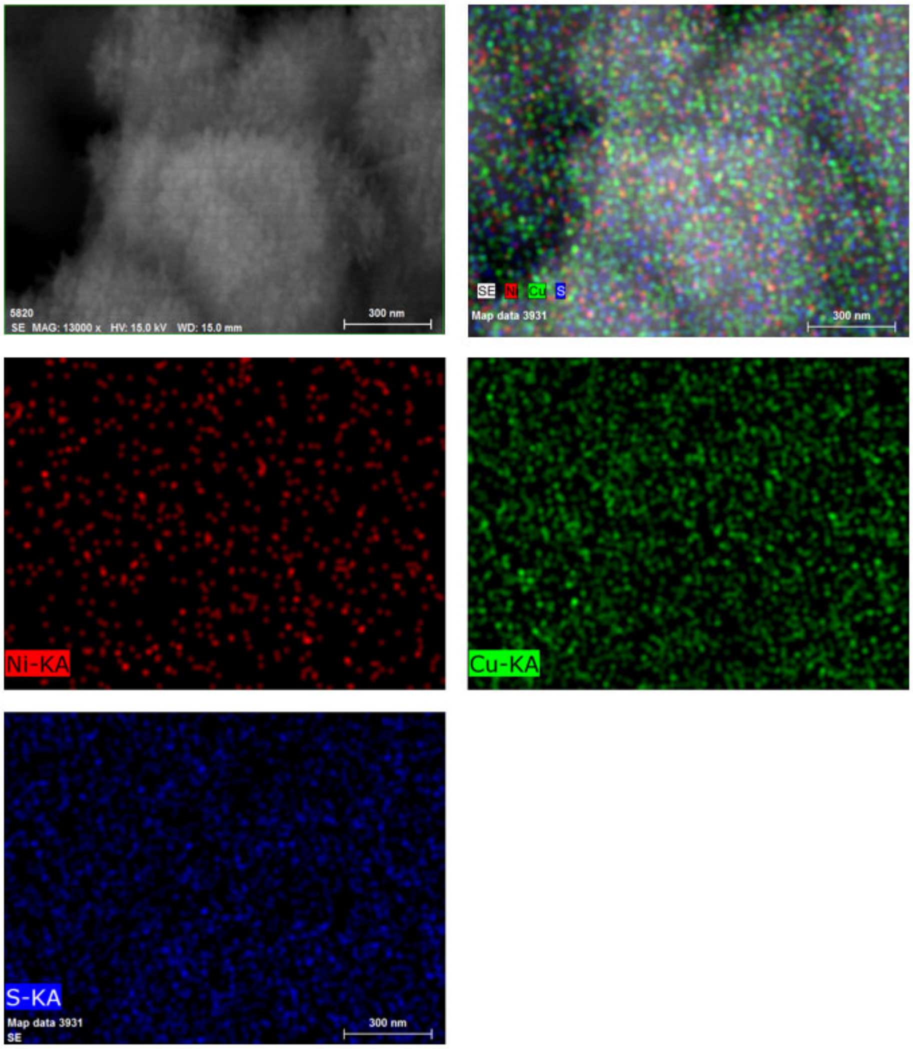

Figure S6. EDS elemental mapping of the 20% NiS-Cu_2S-CF composites.

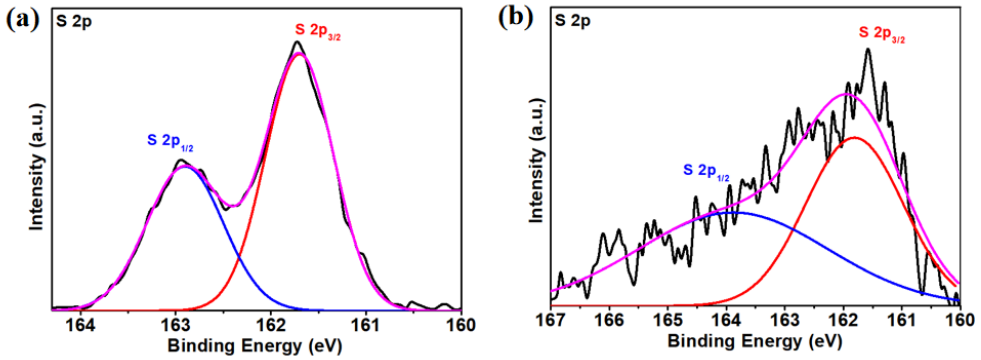

Figure S7. The S XPS spectra of the (a) Cu_2S-CF and (b) NiS-CF composites.

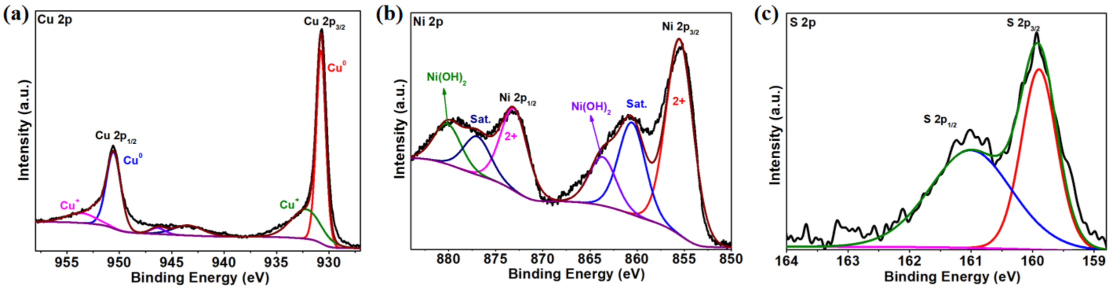

Figure S8. The XPS spectra of the 5% NiS-Cu2S-CF composites, (a) Cu 2p, (b) Ni 2p, and (c) S 2p.

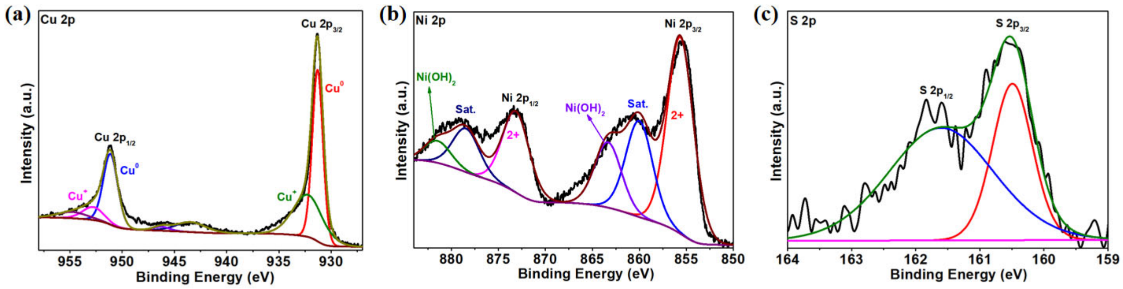

Figure S9. The XPS spectra of the 10% NiS-Cu2S-CF composites, (a) Cu 2p, (b) Ni 2p, and (c) S 2p.

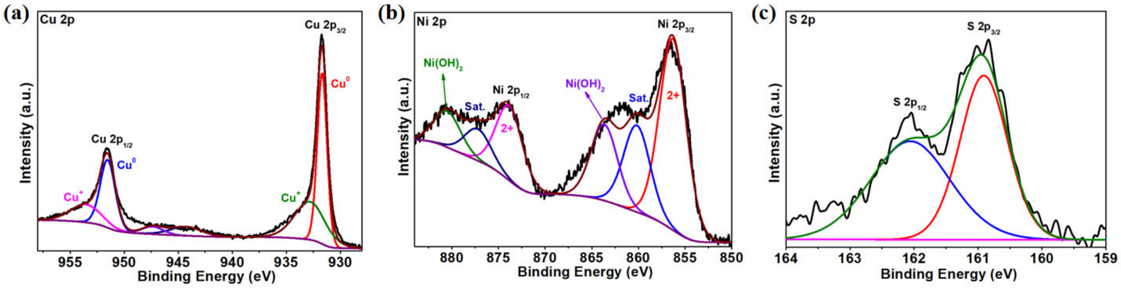

Figure S10. The XPS spectra of the 20% NiS-Cu2S-CF composites, (a) Cu 2p, (b) Ni 2p, and (c) S 2p.

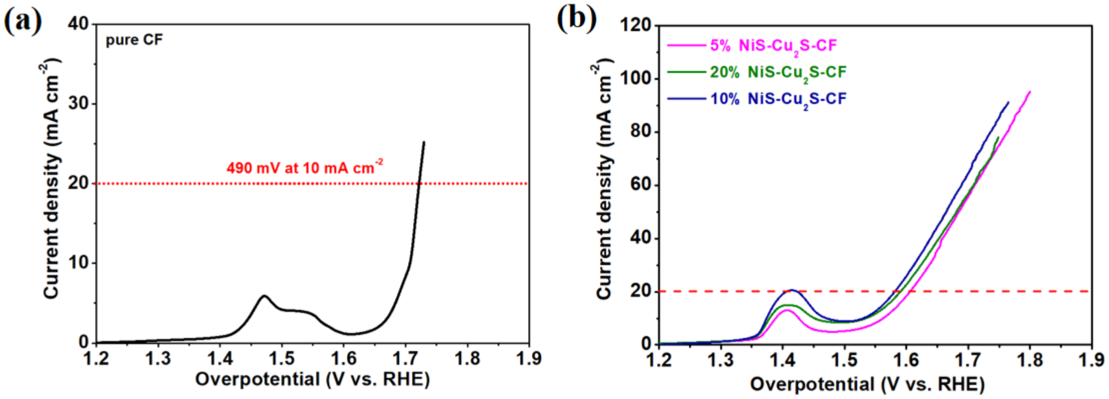

Figure S11. LSV curves of the (a) pure CF and (b) 5% NiS-Cu_2S-CF, 10% NiS-Cu_2S-CF, and 20% NiS-Cu_2S-CF composites.

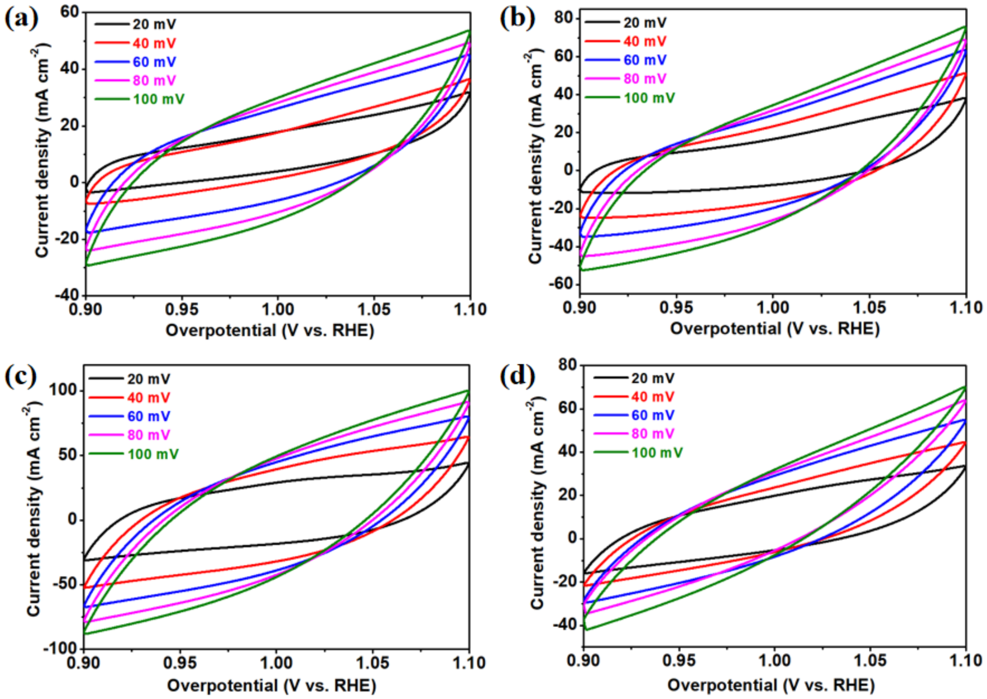

Figure S12. CV curves of the (a) 5% NiS-Cu_2S-CF composites, (b) 10% NiS-Cu_2S-CF composites, (c) 15% NiS-Cu_2S-CF composites, and (d) 20% NiS-Cu_2S-CF composites.

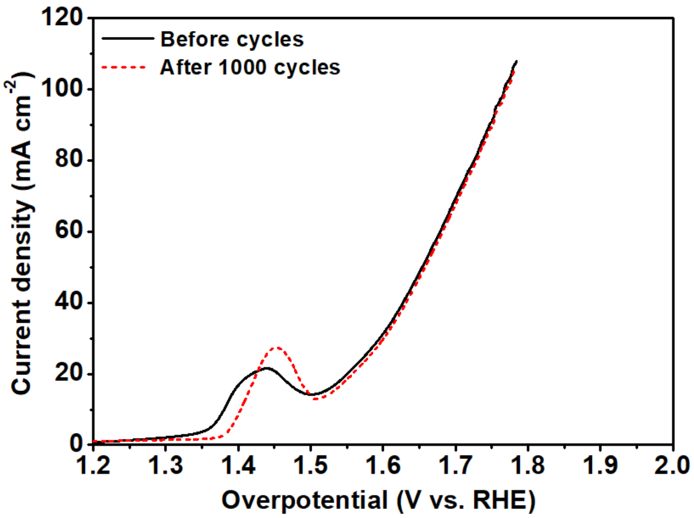

Figure S13. LSV curves of the 15% NiS-Cu_2S-CF composites after 1000 cyclic test.

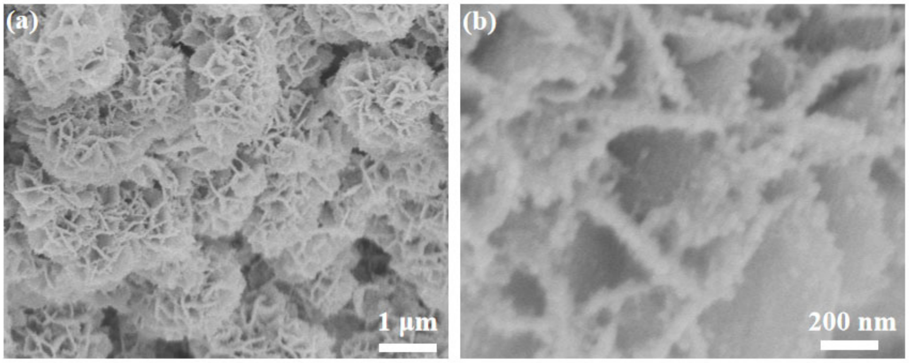

Figure S14. (a,b) SEM images of the 15% NiS-Cu_2S-CF composites after 1000 cyclic test.

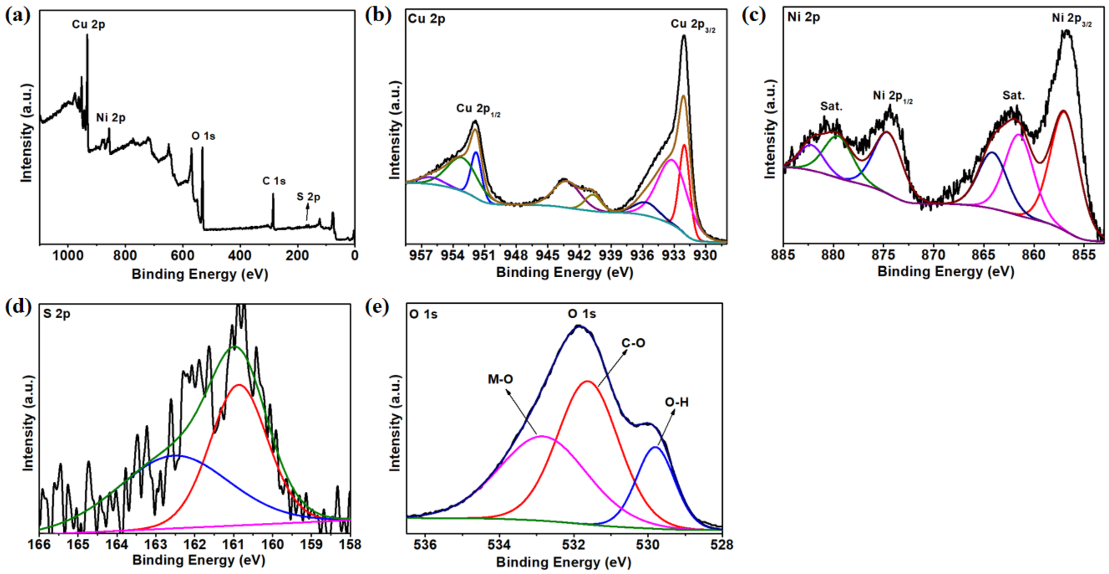

Figure S15. XPS of the 15% NiS-Cu_2S-CF composites after 1000 cyclic test. (a) Fully XPS spectra, (b) Cu 2p, (c) Ni 2p, (d) S 2p, and (e) O 1s.
